# ApoB-Specific CD4^+^ T Cells in Mouse and Human Atherosclerosis

**DOI:** 10.3390/cells10020446

**Published:** 2021-02-19

**Authors:** Timoteo Marchini, Sophie Hansen, Dennis Wolf

**Affiliations:** 1 Department of Cardiology and Angiology I, University Heart Center Freiburg, Hugstetterstraße 55, 79106 Freiburg, Germany; timoteo.marchini@universitaets-herzzentrum.de (T.M.); sophie.hansen@universitaets-herzzentrum.de (S.H.); 2 Faculty of Medicine, University of Freiburg, Breisacherstraße 153, 79110 Freiburg, Germany; 3 Facultad de Farmacia y Bioquímica, Universidad de Buenos Aires, CONICET, Instituto de Bioquímica y Medicina Molecular (IBIMOL), Junín 954, C1113 AAD Buenos Aires, Argentina

**Keywords:** atherosclerosis, immunity, autoimmunity, apolipoprotein B, LDL, T cells

## Abstract

Atherosclerosis is a chronic inflammatory condition of the arterial wall that leads to the formation of vessel-occluding plaques within the subintimal space of middle-sized and larger arteries. While traditionally understood as a myeloid-driven lipid-storage disease, growing evidence suggests that the accumulation of low-density lipoprotein cholesterol (LDL-C) ignites an autoimmune response with CD4^+^ T-helper (T_H_) cells that recognize self-peptides from Apolipoprotein B (ApoB), the core protein of LDL-C. These autoreactive CD4^+^ T cells home to the atherosclerotic plaque, clonally expand, instruct other cells in the plaque, and induce clinical plaque instability. Recent developments in detecting antigen-specific cells at the single cell level have demonstrated that ApoB-reactive CD4^+^ T cells exist in humans and mice. Their phenotypes and functions deviate from classical immunological concepts of distinct and terminally differentiated T_H_ immunity. Instead, ApoB-specific CD4^+^ T cells have a highly plastic phenotype, can acquire several, partially opposing and mixed transcriptional programs simultaneously, and transit from one T_H_ subset into another over time. In this review, we highlight adaptive immune mechanisms in atherosclerosis with a focus on CD4^+^ T cells, introduce novel technologies to detect ApoB-specific CD4^+^ T cells at the single cell level, and discuss the potential impact of ApoB-driven autoimmunity in atherosclerosis.

## 1. Atherosclerosis Is an Immune-Driven, Chronic Inflammatory Disease

Atherosclerosis is a chronic inflammatory disease that is characterized by the build-up of vessel-occluding plaques in the intimal layer of middle- to large-sized arteries [1]. Atherosclerosis precipitates myocardial infarction (MI) and stroke and represents the leading cause of death worldwide [2]. Epidemiologic studies have demonstrated that besides the traditional risk factors of smoking, hypertension, obesity, diabetes, and other environmental factors [3,4], low-density lipoprotein cholesterol (LDL-C) [5,6] is one of the driving factors of developing and progressing atherosclerotic disease. While atherosclerosis was originally perceived as a lipid-storage disease of the arterial wall [7], it is now established that the continuous deposition of LDL-C in the subintimal space is accompanied by a local and systemic low-grade inflammatory and immune response [8]. In the plaque, LDL-C is modified by oxidative processes (oxLDL) and taken up by tissue-resident macrophages. The continuous intracellular accumulation of lipids eventually exceeds the macrophage’s cholesterol storage capacity and intracellular lipid droplets form “foam cells” [9]. Foam cell formation and the per se pro-inflammatory properties of oxLDL [10] initiate a myeloid-dominated immune response with an increasing recruitment of monocytes from the blood circulation [11], a partially self-expanding population of plaque macrophages, and the secretion of proinflammatory cytokines, such as interleukin (IL)-1β by myeloid cells [12]. In addition, a variety of cell types of lymphocytic origin, including B and T cells, accumulate within the plaque and in the surrounding adventitia rendering the cellular architecture of atherosclerotic plaques almost as diverse as that of lymphatic tissue [13]. The continuous secretion of inflammatory mediators from myeloid and lymphoid cells is understood as a self-amplifying inflammatory cascade that ultimately promotes an unstable plaque phenotype, plaque erosion and rupture, and the formation of occlusive arterial thrombi that restrict blood flow and cause critical tissue ischemia in MI and stroke [14]. Of all risk factors, LDL-C provides the strongest causal link between clinical risk and cellular pathology: LDL-C lowering strategies attenuate plaque inflammation, promote plaque regression [15], and have been proven effective in reducing cardiovascular mortality in humans [16]. Likewise, anti-inflammatory treatments targeting the pro-inflammatory cytokine IL-1β [17] and by colchicine [18,19] prevent the progression of cardiovascular disease. A growing body of evidence suggests that the inflammatory milieu in the plaque is accompanied by a powerful autoimmune response [20,21] involving auto-reactive CD4^+^ T cells [22] and autoantibodies secreted by B cells [23]. While additional autoantigens cannot be excluded, overwhelming evidence shows that LDL-C represents the main culprit of this autoimmune response: While most auto-antibodies are directed against oxidation-specific epitopes in the lipid surface of LDL [23,24], autoreactive T cells recognize peptides from Apolipoprotein B (ApoB) [20], the core protein of LDL-C. Atherosclerosis can therefore be understood as chronic inflammatory disease of the cardiovascular system with a significant autoimmune component [21]. Here, we focus on the role of autoreactive CD4^+^ T-helper cells and comment on necessary developments to successfully translate novel immunomodulatory strategies into clinical practice.

## 2. Frequencies, Immune Phenotypes, and Roles of T-Helper Cells in Atherosclerosis

T cells represent the largest and most heterogeneous leukocyte population in human atherosclerotic plaques [25]. In immunohistochemistry (IHC), CD4^+^ T-helper and CD8^+^ cytotoxic T cells are detected in the shoulder region, the fibrous cap, and the intima of the plaque as well as in adventitial tissue [26]. In human plaques, T cells account for more than 50% of all lesional leukocytes with the highest T cell density in the shoulder region, while T cells account for less than 15% in the macrophage-dominated necrotic core [13,25,26,27]. In single cell RNA-sequencing (scRNAseq) and mass cytometry by time of flight (CyTOF) of human atherosclerotic plaques, T cells outnumber other hematopoietic lineages and reach a frequency of up to 65% of all leukocytes [28,29]. In atherosclerotic plaques from mice, T cell are less frequent and—depending on the underlying genetic model—range between 6% and 25% of all leukocytes [13,30,31]. In contrast to IHC, protocols employing tissue digestion and cell isolation are at the risk of overestimating non-myeloid cells due to a potential loss of macrophages during tissue digestion [13,25]. Notably, healthy arterial tissue contains CD8^+^ T cells in small frequencies [13]. Among T cells, CD4^+^ and CD8^+^ T cells are found in similar frequencies in atherosclerotic mouse aortas and human atherosclerotic plaques [25,28].

T cells develop from T cell precursors in the thymus. They transit through different developmental stages, including CD4^+^CD8^+^ double-positive (DP) T cells, and turn into either cytotoxic CD8^+^CD4^−^ or helper CD4^+^CD8^−^ T cells [32]. All T cells express CD3 and a unique T cell receptor (TCR) that binds antigenic peptides loaded on major histocompatibility complex (MHC): CD8^+^ T cells recognize peptides from intracellular proteins that are degraded by cytosolic and nuclear proteasomes and loaded on MHC-I, which is expressed in all nucleated cells [33]; CD4^+^ T cells recognize peptides from extracellular proteins that are taken-up via several pathways, degraded in the endosome, and loaded on MHC-II, which is primarily expressed in antigen presenting cells (APCs), such as dendritic cells (DCs) or plaque macrophages [33]. After the MHC-peptide complex is bound by their TCR, T cells are activated and develop into effector T cells (T_eff_). Antigen-recognition and co-stimulation are central processes for the differentiation and activation of CD4^+^ T-helper cells (T_H_) [34]. T_eff_ develop into functionally and phenotypically distinct types of T_H_ immunity that are characterized by the expression of canonical intracellular transcription factors (TF) and cytokines. Solid evidence, mostly from preclinical mouse studies, has established a distinct, but often controversial, role for many known T_H_-lineages in atherosclerosis.

### 2.1. T_H_1 Cells

Studies of CD4^+^ T cells in human plaques have suggested that—depending on cytokine secretion patterns—30–70% of all CD4^+^ T cells share features with T_H_1 cells [35,36]. T_H_1 cells express the TF T-bet [37], and secrete Interferon (INF)-γ, IL-2, IL-3, Tumor necrosis factor (TNF), and lymphotoxin [20,35,37]. In general immunity, T_H_1 CD4^+^ T cells are critical for developing an immune response against pathogens by enhancing the microbicidal activity of macrophages through enhanced IFN-γ production [38]. T cells from atherosclerosis-prone Apolipoprotein E deficient (*Apoe*^−/−^) mice secrete IFN-γ [39,40]. A genetic deficiency of IFN-γ [41,42], its receptor [43], and of T-bet [44] protects from atherosclerosis. Consistently, IFN-γ administration aggravates atherosclerosis in mice [45]. C-C chemokine receptor (CCR)-5 serves as homing receptor for T_H_1 cells [37] and is upregulated in plaque T-helper cells [28]. A genetic deficiency of CCR5 protects from T cell homing into the plaque in mice [40]. Therefore, T_H_1 cells are generally regarded as pro-atherogenic cells [20].

### 2.2. T Regulatory Cells

T-regulatory cells (T_reg_) are defined by the expression of the transcription factor FoxP3 and IL-2 receptor (CD25). T_regs_ are required to maintain self-tolerance and dampen immunity by secreting the immunosuppressive cytokine IL-10, Transforming growth factor (TGF)-β, and by direct contact-inhibition of T_eff_ cells [46,47]. T_regs_ are found in human atherosclerotic lesions at a frequency of 1.2–3.9% of all CD3^+^ T cells [48]. In the mouse, 5% to 10% of CD4^+^ T cells express the T_reg_-lineage defining TF FoxP3 suggestive of T_regs_ [49,50]. T_regs_ express high levels of CTLA4, GITR, and lack expression of CD127 in humans, which enables the detection and cell sorting of viable T_regs_ [47,51,52,53]. In mouse atherosclerosis, T_regs_ are generally regarded as protective [54,55] and expand in regressing plaques [56]. Accordingly, IL-10 deficiency promotes atherosclerosis in mice [46]. Clinically, blood T_reg_ numbers and IL-10 plasma levels are lower in patients with MI compared with healthy individuals [57]. A low fraction of T_reg_ among all CD4^+^ T cells predicts MI [58]. However, the regulation and function of FoxP3^+^ T_regs_ in atherosclerosis remains controversial: In humans, one report has suggested higher frequencies of circulating T_regs_ in patients with stable atherosclerosis compared with healthy controls [49]. In mice, the population of T_regs_ in the spleen [59,60] and the liver [61] of *Apoe*^−/−^ and *Ldlr*^−/−^ mice increases in the context of hypercholesterolemia. These associative findings argue against a solely protective role of T_regs_ and may be partially explained by the appearance of T_reg_-like CD4^+^ T cells that express FoxP3 and pro-inflammatory cytokines in advanced atherosclerosis [37,40,49]. The function of these abnormal T_regs_ will be discussed below. 

### 2.3. T_H_17 Cells

T_H_17 cells express the TF RORγT and secrete IL-17 [62]. T_H_17 CD4^+^ T cells are gatekeepers of mucosal immunity and have been associated with several autoimmune diseases. They are activated by IL-23 and secrete the cytokines IL-17A and -F [63]. Numerous studies have revealed a highly controversial role of T_H_17 cells in mouse and human atherosclerosis: Some studies showed proatherogenic effects [64,65,66,67] in mice and higher plasma IL-17 levels in humans with unstable angina or MI [68,69]. These findings are consistent with an overall proatherogenic role of T_H_17 immunity [27]. Other studies demonstrated atheroprotective and plaque-stabilizing properties in mice [70,71,72,73,74] and lower plasma levels of IL-17 in patients with acute MI [75] suggestive of an overall atheroprotective role [41]. In addition, some studies have found no role for T_H_17 cells in mice [76], which is in accord with unchanged IL-17 plasma levels in humans with or without coronary artery disease (CAD) [77]. 

### 2.4. T_H_2 Cells

T_H_2 cells are involved in the immune response against parasites, in asthma, and other allergic diseases [78]. They express the TF Gata3 and secrete IL-4, IL-5, IL-10, and IL-13 [13]. Their role in atherosclerosis is unclear. Pro-atherogenic and atheroprotective functions have been proposed in mice [79,80,81,82]. In humans, low T_H_2 cell numbers and weak IL-4 secretion from CD4^+^ T cells predict myocardial infarction [83]. In addition, low plasma concentrations of IL-5 associate with subclinical carotid atherosclerosis [84]. Both studies argue for a protective role of T_H_2 immunity in progressing and de novo atherosclerosis. Likewise, IL-33 administration reduces murine atherosclerosis by increasing levels of IL-4, IL-5, IL-13, and INF-γ in the plasma [85]. 

### 2.5. Follicular-Helper T Cells (T_FH_)

T-follicular helper cells (T_FH_) provide help for B cells and are required for the antibody isotype switch in germinal center B cells [86]. They express the TF Bcl-6 and the chemokine receptor CXCR5 [86]. Dyslipidemia in *Apoe*^−/−^ and *Ldlr*^−/−^ mice induces T_FH_ cells and IgG2c production [87]. Depletion of T_FH_ cells protects from atherosclerosis [50]. T_FH_ cells have been suggested to orchestrate a pro-atherogenic B cell response in mouse atherosclerosis [88]. Ageing increases the number of T_FH_ cells in *Apoe*^−/−^ mice [89]. T_FH_ cells are found more frequently in advanced atherosclerosis [90]. It has been suggested that T_FH_ cells are at least partially derived from T_reg_ cells [50] and from ApoB-specific CD4^+^ T cells [91].

### 2.6. CD4^+^ Cytotoxic Lymphocytes (CTL)

Lymphocytes with a cytotoxic potential include natural killer (NK) cells, CD8^+^ T cells, NK T cells, γ/δ T cells, and a subset of human CD4^+^ T cells that is characterized by a down-regulation of the co-stimulatory molecule CD28 (CD4^+^CD28^null^ T cells) [92]. CD4^+^ CTLs represent a highly differentiated subset of memory T cells [93], secrete perforin, granzyme A and B, and TNF-α and IFN-γ [94] and express high levels of the exhaustion marker OX-40 [95]. They have not been detected in mice [96] and are found in human vulnerable atherosclerotic lesions [97,98,99]. Their distinct function in atherosclerosis has not been tested so far [94]. It may be that CD4^+^ CTLs do not represent a distinct T-helper cell lineage, but instead the fraction of antigen-specific [100,101], terminally differentiated, and exhausted CD4^+^ T cells that acquire cytotoxic functions [28,102]. 

### 2.7. Other Types of T Cell Immunity

T_H_9 are characterized by the expression of IL-9 in response to TGF-β and IL-4. Their generation is inhibited by INF-γ [103] and depends on several TF, including FoxO1, BATF, and IRF4 [104]. Clinically, IL-9 plasma levels are higher in patients with atherosclerosis [105] and with an acute coronary syndrome, while the count of T_H_9 cells was unchanged in another study [106]. IL-9 administration seems to promote atherosclerosis in *Apoe*^−/−^ mice [107], but the overall role of T_H_9 cells remains unknown. T_H_22 cells express IL-22 and the TF aryl hydrocarbon receptor (AHR) [108]. Based on one report, T_H_22 immunity may be pro-atherogenic [109]. T_H_22 cells in the blood [106,110] and plasma levels of IL-22 are increased in patients with an acute coronary syndrome [106,110]. Other T cell subsets described in atherosclerosis include innate-like NK T cells that express restricted pairs of TCR α- and β- chains and recognize self and foreign lipid antigens presented on the MHC-I like molecules CD1d [111]. NK T cells secrete T_H_1, T_H2_, and T_H_17 cytokines as well as perforin and granzyme B. NKT cells are found in rupture-prone human atherosclerotic plaques [112], but their role in atherosclerosis remains controversial [94,113]. CD8^+^ T cells represent the main cytotoxic T cell subset that participates in the immune defense against intracellular pathogens and in tumor surveillance [114]. In atherosclerosis, CD8^+^ T cells have been attributed to a multitude of both pro- and anti-inflammatory roles. CD8^+^ T cells may suppress inflammation, control macrophage accumulation, and partially by direct cytotoxic effects on lesional macrophages, contribute to endothelial cell surveillance and damage, and exhibit direct effects on myelopoiesis. Whether CD8^+^ T cells are antigen-specific is a matter of debate. The roles of CD8^+^ T cells has been extensively reviewed [115] and is beyond the scope of the present review.

### 2.8. Multi-T_H_ Committed CD4^+^ T Cells in the Atherosclerotic Plaque

Traditionally, T_H_-types in CD4^+^ T cells represent a unidirectional and terminal path of differentiation that is inflexible and irreversible. This concept has been challenged by growing evidence that T_H_-cells can reprogram towards mixed phenotypes of T_H_ cells or re-differentiate into alternative T_H_ types of cells [116]. T_regs_ and T_H_17 cells seem to be particularly prone to such T_H_ cell plasticity [117]: T_regs_ can acquire features of T_H_1 (T_H_1-T_regs_) or T_H_17 cells (T_H_17-T_regs_), or switch into T_FH_ cells [37,40,118,119,120,121,122,123]. Mechanistically, it has been demonstrated that initially immunosuppressive FoxP3^+^ T_regs_ downregulate FoxP3 protein expression in direct lineage tracing experiments and give rise to exT_regs_ that express alternative TFs [49,50]. In the atherosclerotic plaque, a considerable fraction of CD4^+^ T cells express low levels of FoxP3 as well as of IFN-γ, and T-bet [37,40]. These cells promote atherosclerosis after an adoptive transfer, have lost their immunosuppressive properties, and act as effector T cells [40]. Consistently, recent scRNAseq of T cells from mouse atherosclerotic plaques demonstrated CD4^+^ T cell clusters with mixed T_H_1/T_H_2/T_reg_ and T_H_1/T_H_17 transcriptomes that account for approximately 50% of all lesional T cells [49]. The co-expression of genes suggestive of T_H_1- and T_H_17-phenotypes has also been demonstrated in CD4^+^ T cells isolated from human carotid plaques [28]. In single cell gene module enrichment analysis, core genes of these mixed phenotypes were enriched in tetramer-selected ApoB-specific CD4^+^ T cells [49], proposing that T_reg_ lineage instability occurs frequently in antigen-specific T cells in the plaque. In addition to multi-T_H_ committed CD4^+^ T cells in atherosclerotic plaques, the existence of IL-17 or RORγT expressing T_regs_ has also been suggested in the blood of patients with cardiovascular disease (CVD) [49,50]. These findings indicate that the generation of CD4^+^ T cells with features reminiscent of several T_H_-types is a systemic, rather than a local event in atherosclerosis. Together, these findings question whether traditional T_H_ lineages reflect the actual functional heterogeneity of T-helper cells in the atherosclerotic plaque (Table 1). Whether such high plasticity is driven by antigen-specificity as suggested by other disease models beyond atherosclerosis [124,125,126] will be discussed below.

## 3. Evidence for Autoimmunity in Atherosclerosis

Autoimmune disease is defined as an abnormal response of the immune system against endogenous proteins and other components of the body (autoreactivity). This immune response can lead to the damage, destruction, or functional loss of involved tissues [130]. Naturally occurring T_regs_ prevent autoimmunity against self-peptides and -antigens [47]. As outlined above, the accumulation of T cells in the plaque and the appearance of circulating autoantibodies in patients with atherosclerosis has inspired the idea of an autoimmune component in addition to antigen-independent inflammation [131]. Several observations support this hypothesis: First, T cells in the plaque of mice and humans exhibit an unexpected strong memory phenotype with hallmarks of chronic stimulation and T cell exhaustion as evidenced by a high proportion of CD4^+^CD45RA^low^CCR7^low^ T_EM_ and of CD4^+^ T cells expressing high levels of the activation markers CD69 and CD38 [28] and of cytotoxic factors [29]. These findings suggest the presence of antigen-specific T cells that have built up a T cell memory against antigens that are likely present in the plaque or the draining lymphatics [28,60]. The expression of programmed cell death protein 1 (PD-1), a known exhaustion marker, and of several genes associated with exhaustive T cell signaling, such as *EOMES* and *LAG3*, in human plaques further argues for chronic antigen recognition by CD4^+^ T cells in the plaque [28]. T cell exhaustion is understood as a negative regulator and checkpoint of chronic T cell stimulation and -antigen recognition [132]. Inhibition of PD-1, which dampens subsequent cellular activation, results in aggravated atherosclerotic disease in mice [133]. This finding is consistent with the hypothesis that antigen-specific and pathogenic CD4^+^ T cells in the advanced plaque become resistant to the ongoing recognition of their cognate antigens by exhaustive transcriptional programs. A therapeutic checkpoint inhibition, as performed in several malignancies, could therefore re-activate plaque T cells, and provoke complicated atherosclerosis [28,134]. Second, T cells frequently interact with plaque-resident APCs in live cell imaging in mice, in particular when APCs and T cells originate from atherosclerotic and hypercholesterolemic *Apoe*^−/−^ mice. As result of this physical interaction, T cells secrete pro-atherogenic cytokines such as IFN-γ [39]. Notably, pro-inflammatory cytokine secretion in this model requires the presence of atherosclerosis-related antigens and is not observed when unrelated model antigens are used as control. Likewise, human and mouse T cells from atherosclerotic plaques secrete cytokines and proliferate when restimulated with LDL or peptides from ApoB [22,49,135]. Third, T cell activation is a result of antigen-recognition and promotes the proliferation of antigen-specific T cells with the same TCR to build oligoclonal populations. In mouse plaques, T cell proliferation is evident in histological analysis [39,136] and in scRNAseq, where clusters of proliferating cells contained T cell signatures [13,137]. Lesional T cells seem to be clonally enriched in TCR-sequencing [138] in mice. In vitro cloned T cell lines reactive against ApoB show a preferential usage of the V-chain segment 31 (TCRBV-31) [135]. Consistently, MHC-II tetramer selected T cells expressed an oligoclonal TCR-repertoire [49]. In humans, T cell clonality in the plaque [97,139] and in coronary thrombi [140] are restricted in TCR-usage. Only one report has suggested a non-restricted repertoire in atherosclerotic aortas [141]. Whether CD8^+^ or CD4^+^ T cells or both are the cause of such TCR-restriction is not known, but a recent report has demonstrated a correlation between T cell exhaustion in lesional CD8^+^ T cells and TCR-clonality [28]. Fourth, several autoantigens have been derived from direct vaccination experiments: LDL-C/ApoB (discussed below), heat shock proteins (HSPs) [142,143], and β2-Glycoprotein I (β2GPI). HSPs are intracellular, highly species-conserved chaperones that protect against stress and physical irritation such as temperature, UV light, and changes in the pH [144]. In humans, antibodies directed against HSP60 correlate with cardiovascular disease [145,146]. In addition, immunization using HSP60/65 and peptides thereof as antigens modulates atherosclerosis [147,148,149,150,151,152,153,154,155,156]. Interestingly, it was proposed that bacteria derived HSP65 induces an autoreactive response against human HSP60. Both molecules express similar immunodominant B cell epitopes [157], which may explain a cross-reactivity between infection-derived epitopes and self-epitopes as recently shown for the cross-reactivity between *Streptoccocus pneuomniae* and oxidation-specific epitopes in mice [158]. β2GPI is the target of anti-cardiolipin antibodies [159] that cause the anti-phospholipid syndrome, a state of hyper-coagulation in systemic lupus erythematosus patients [160]. β2GPI has been found in human atherosclerotic lesions [161] but direct vaccination experiments using β2GPI have yielded inconsistent results [162,163,164,165,166]. In addition to the aforementioned autoantigens, several targets of IgM- and IgG-autoantibodies, mostly oxidation-specific epitopes of LDL, have been suggested [23,24,167]. It has also been discussed that a fraction of antigen-specific T cells in the atherosclerotic plaque recognizes infectious peptides from bacteria or viruses. This hypothesis is based on numerous observations, foremost the clinical association of infectious disease and atherosclerosis: For instance, observational studies have established that an infection with Varicella Zoster Virus (VZV) and Influenza Virus increases the risk for MI and stroke [168,169]. Vaccination against Influenza is now recommended for secondary prevention of patients with heart disease [170] and improves the cardiovascular outcomes [171,172,173]. Human Cytomegalovirus (HCMV), Herpes Simplex (HSV), Epstein Barr Virus (EBV), VZV, and Influenza Virus were suspected of causing an infection of the arterial vessel wall [174,175,176]. However, only in rare cases, viral particles have been detected within atherosclerotic lesions [176,177,178] and a potential interference of infection and atherosclerosis may be explained by increased inflammatory signaling cascades, local tissue injury, and enhanced thrombotic pathways [176,179] rather than a direct pathogenicity of virus-specific T cells in the atherosclerotic plaque. Such indirect effects also seem to trigger some of the cardiovascular complications of SARS-CoV2 [180]. In addition, it cannot be excluded that some autoreactive T cells in the plaque are cross-reactive to exogenous infection with a structural similarity (molecular mimicry) to autoantigens as shown for *S. pneumoniae* which induces antibodies that bind oxLDL [158,181,182]. Whether pneumococcal vaccination is beneficial in targeting auto-antigens in the plaque remains controversial [183,184,185]. Of all proposed atherosclerosis-related (auto-) antigens, LDL-C and ApoB provide the strongest causal link between autoimmunity and the pathogenesis of atherosclerosis. We will therefore focus on the role of ApoB-specific CD4^+^ T-helper cells in the following sections.

## 4. ApoB-Specific CD4^+^ T Cells in Mice and Humans

### 4.1. Mechanisms of CD4^+^ T Cell Activation

The activation and transition from naïve to effector/memory T cells (T_EM_) is a process that encompasses two signals: The presentation of the antigen by an APC (Signal 1) and additional co-stimulatory signals (Signal 2). DCs and macrophages are found in healthy arteries and atherosclerotic plaques [8,131,186] and serve as APCs at different stages of the disease [187]. Cellular, sub-cellular, or molecular antigens are taken up by the APC by phagocytosis or endocytosis and processed in the endosome. Antigen-derived peptides may then bind to an MHC in the Golgi apparatus depending on the binding affinity between the MHC and the peptide. The complex of peptide and MHC is next translocated to the cell surface [188]. In the presence of a suited antigen-specific T cell, the MHC-peptide complex is bound by a unique TCR. The bond between TCR and MHC-peptide is stable for several hours [189]. TCR signal-transduction in the T cell is mediated by the complex of CD3, CD4, and the TCR [190], and by down-stream signaling molecules like zeta-chain-associated protein kinase 70 (ZAP-70) and SH2 Domain-containing Leukocyte Protein of 76 KDa (SLP-76) [191]. These signaling events result in the transcription and translation of proteins necessary for the differentiation and proliferation of the activated T cell [192,193]. “Signal 2” describes the additional signaling by a total of 38 possible combinations of co-stimulatory and co-inhibitory ligands and receptors [194]: Co-stimulatory pairs of ligands and receptors, such as CD28/CD80, promote activation whereas others, such as CTLA4 and CD80, prevent subsequent TCR-signaling and cell activation [195,196,197]. TFs and signaling pathways in APCs that induce a tolerogenic response in T cells include IL-10, TGF-β, Flt3-, and Myd88-dependent signaling events [198,199,200,201], while cholesterol accumulation and IRF-8-dependent signaling promote an immunogenic response [202,203]. Further differentiation signals are provided by pro- or anti-inflammatory cytokines secreted by APCs [186,204,205,206]: The anti-inflammatory signals IL-10 and TGF-β induce tolerogenic responses and predispose to T_reg_ differentiation, while IL-6 prompts a T_H_17, and IL-12 a T_H_1 response. A firm cellular bond between the APC and the T cell requires the interaction of additional cell–cell adhesion molecules, such as LFA-1/ICAM [207]. The physical interaction site of the T cell and the APC that exhibits a high density of cell adhesion molecules, co-stimulatory molecules, and peptide-loaded MHC/TCR complexes is often referred to as the immunological synapse [208]. Several assays have been designed to identify antigen-specific T cells, including single cell detection by MHC-II multimers, functional restimulation, and cloning of T cell lines [209,210,211].

### 4.2. Detection of ApoB-Specific CD4^+^ T Cells in Humans by Functional Restimulation

**Figure 1:**
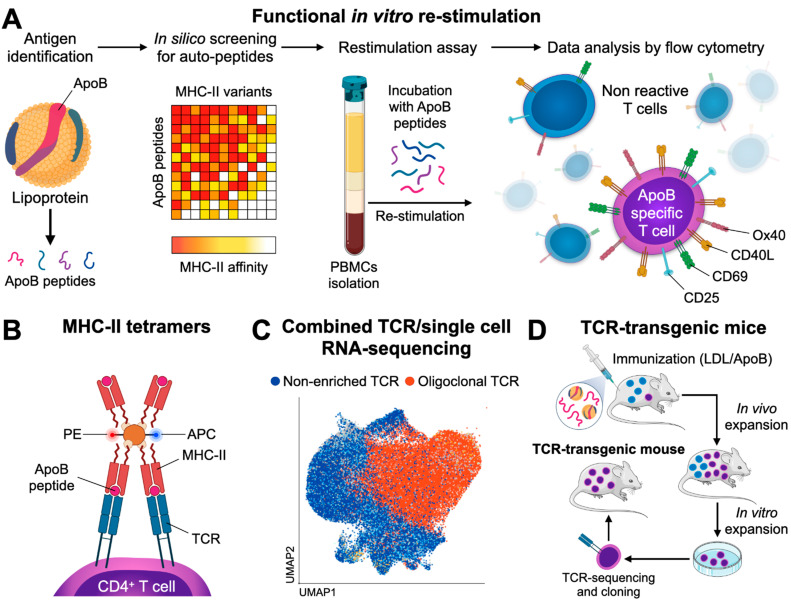
Approaches to detect Apolipoprotein B (ApoB)-specific CD4^+^ T cells in mice and humans. Functional restimulation with ApoB-peptides requires a peptide mapping of the entire ApoB-sequence and a selection of peptides with the highest predicted and tested affinity towards the MHC-II complex. Antigen-presenting cells are loaded with selected peptides and co-incubated with CD4^+^ T cells. Expression of activation markers is subsequently used to identify activated, ApoB-reactive T cells (**A**) Design of tetramers of MHC-II loaded with the peptide of interest. Tetramers are labelled with fluorochromes for the detection of tetramer-binding T cells in flow cytometry. (**B**) In simultaneous T cell receptor (TCR) and gene expression RNA-sequencing, T cells with an oligoclonal repertoire of the TCR are detected on a single cell level, which allows to selectively analyze gene expression in these cells. (**C**) Workflow for the generation of a TCR-transgenic mouse: peptide-specific T cells are expanded in vivo by immunization with LDL or a self-peptide from ApoB, isolated, and further expanded ex vivo by restimulation with LDL or peptide-loaded APC. Resulting clones undergo TCR-sequencing and the most promising TCR-sequences are used as templates for the generation of a TCR-transgenic mouse (**D**).

We have recently introduced an in vitro restimulation assay for the detection of ApoB-specific CD4^+^ T cells in humans [49] (Figure 1A). In this assay, human PBMCs including APCs and T cells are co-incubated in vitro with a mix of antigenic peptides from ApoB-100. To limit all possible ApoB-100 peptides to the ones that could potentially be loaded on MHC-II, an in-silico screening of human ApoB-100 and direct MHC-II-peptide affinity measurements was performed. This screening strategy generated a pool of ApoB-100 candidate peptides with a high affinity for several human MHC-II alleles, thereby covering 80% of a Caucasian population with unknown MHC-II variants [49]. The upregulation and detection of T cell activation markers as a result of peptide-recognition by T cells serves as marker for peptide-specific CD4^+^ T cells. In vitro culturing itself is known to decrease cell viability and interferes with T cell differentiation pathways, TF expression, and cytokine secretion. Therefore, the time of restimulation has to be kept to a minimum. Accordingly, the kinetics of cell surface marker expression used for the identification of activated CD4^+^ T cells needs to be carefully considered. CD25, CD69, CD154 (CD40L), and OX40 are established CD4^+^ T cell activation markers [212] and are highly expressed in human unstable atherosclerotic plaques [213]. CD25 is the receptor for IL-2 (IL-2R) and peaks 72 h after CD4^+^ T cell stimulation. CD25 is therefore not suited as an immediate activation marker [214]. OX40 shows similar dynamics and peaks between one and five days after stimulation [215]. Contrastingly, CD69 is upregulated already 30 to 60 min after stimulation with a sharp decrease in expression after 4 to 6 h. Comparative studies have shown that frequencies of antigen specific T cells found after short time stimulation using CD69 and CD40L were similar compared with a stimulation for 8 h using CD25 and OX40 as activation markers [216]. Although its expression profile seems most favorable for in vitro activation assays, CD69 is expressed on naïve and memory T cell subsets and responds to unspecific cellular activation, such as by calcium ionophores. Contrastingly, CD40L has been shown to serve as an immediate activation marker with a high specificity for TCR-signaling events [217], making it an ideal candidate for antigen-specific restimulation assays. A downside of using CD40L remains its transient extracellular expression [218]: After translocation to the cell surface, CD40L is quickly degraded, likely by matrix-metalloproteinases [219], and internalized [220]. We have validated an in vitro assay for the identification of human ApoB-100 specific T-cells employing intracellular CD40L as immediate activation marker in a restimulation assay for 6 h [49]. Using this assay, we were able to show that T cells specific for ApoB-100 peptides exist in the blood circulation of patients with CAD, but not in healthy individuals. In this assay, patients with CAD expressed higher levels of TNF-α, IFN-γ, and IL-17. Contrastingly, the expression of IL-10 decreased in patients with CAD compared to healthy individuals. The findings demonstrate that T-helper cells specific for several ApoB self-peptides exist in humans with atherosclerotic disease.

### 4.3. Detection of ApoB-Specific CD4^+^ T Cells in Mice and Humans by Tetramers of MHC-II

Kimura et al. recently introduced multimers of MHC-II loaded with ApoB-specific peptides to detect peptide-reactive CD4^+^ T cells at the single cell level [118]. These reagents take advantage of the binding of recombinant MHC-II molecules with a pre-defined peptide to a TCR solely specific for this MHC-II-peptide complex. Because the MHC-II-peptide–TCR binding of monomeric MHC-II complexes is weak with a short half-life in the range of seconds, the avidity of this interaction can be increased by coupling several MHC-II-peptide complexes that engage more than one TCR, often as tetramers or dextramers [209,210,211]. The labeling of these reagents with classical fluorochromes for flow cytometry allows the subsequent detection of T cells specifically binding this MHC-II-peptide complex, i.e., T cells with a TCR specific for this (peptide) antigen (Figure 1B). Kimura et al. made use of a combined in silico screening for the 28 most common human MHC-II (HLA-DR) alleles and verification of affinities in competitive binding assays. Several peptide sequences of human ApoB were identified and incorporated into corresponding tetramers. In a sub-study of participants of the Women’s Interagency HIV Study (WIHS) expressing the HLA-DRB1*07:01 allele, 0.17% of all CD4^+^ T cells were reactive against the peptide-epitope p18 (SLFFSAQPFEITAST). More than half of all ApoB/p18-reactive T cells from donors without atherosclerotic disease expressed FoxP3, which indicates the predominance of an immunosuppressive phenotype. The remaining ApoB/p18-reactive T cells expressed RORγt, Gata3, or T-bet. TF expression in ApoB:p18 specific T cells was not exclusive and often occurred in combinations, indicating the existence of T_H_17-T_regs_ and T_H_1-T_regs_. Notably, the fraction of these multi-lineage committed cells increased, in particular the co-expression of the T_H_1 TF T-bet, in donors with subclinical atherosclerosis, while the fraction of single FoxP3 expressors decreased. These results therefore suggest that ApoB-specific CD4^+^ T cells in humans shift towards a more inflammatory phenotype in the context of atherosclerosis. In another study, we recently introduced a tetramer of mouse MHC-II (I-A^b^) to characterize mouse CD4^+^ T-helper cells recognizing the ApoB peptide p6 (ApoB_978–993_) [49]. ApoB-reactive T cells isolated with this tetramer were detectable in lymph nodes of healthy and atherosclerotic mice and showed a predominant T_reg_ and partially atheroprotective phenotype in healthy *Apoe*^−/−^ mice. Notably, these cells had formed a memory phenotype already in healthy mice in about 20% of all ApoB^+^ T cells. In the setting of hypercholesterolemia, ApoB^+^ T cells proliferated, expressed pro-inflammatory genes, partially lost the T_reg_-defining TF FoxP3, and converted into pathogenic T_H_1 and T_H_17-like cells with an only residual T_reg_ gene signature. Both studies demonstrate that tetramers represent a feasible method to detect ApoB-specific CD4^+^ T cells in human blood and in murine lymphoid tissue. It is noteworthy to point out that tetramers with a single peptide specificity may underestimate other T cells clones binding overlapping, adjacent sequences, and that TCR-binding to a given peptide sequence may be less specific, as supposed in [181]. Together with our recent observation that ApoB-specific T cells overlapped with a fraction of 50% of lesional T cells in atherosclerotic aortas [49], it is highly plausible that T cells with more peptide specificities against ApoB or other autoantigens exist in atherosclerotic plaques. Because TCR-clonality in the aorta may directly link to a functional phenotype as recently suggested [221], combined TCR-sequencing and single cell RNA-sequencing workflows may provide a valuable tool to directly infer antigen-specificity from TCR-clonality in scRNAseq in future (Figure 1C). Although in situ tetramer staining in tissue sections is technically possible [222], it has not been tested on atherosclerotic plaques yet.

### 4.4. Cloning of CD4^+^ T Cell Lines and TCR-Transgenic Mice

In the naïve organism, only a small fraction of T cells is expected to be specific for a given (auto) antigen. The absolute size of a population of T cells specific for self or foreign antigens differs considerably and ranges between 10 and 10,000 cells per mouse [181]. Around ~1200 CD4^+^ T cells specific for the self-peptide p6 are found in *Apoe*^−/−^ mice [49]. These numbers render it experimentally extremely difficult to assess the function of ApoB^+^ T cells in in vivo. Recently, Gistera et al. have reported the first TCR-transgenic mouse recognizing human LDL/ApoB [91]. In a series of reports (Figure 1D), the authors first isolated CD4^+^ T cells from transgenic mice expressing human ApoB100 that were immunized with human oxLDL. T cells from these mice were isolated, restimulated with human oxLDL, native human LDL, or purified human ApoB-100, and reactive single cell clones were identified by IL-2 expression in vitro. V-segments of the T α- and β-chains of the TCR were determined by RT-PCR on IL-2 reactive clones. The TCR-β V-segment 31 was the only V-β segment uniformly expressed across all clones. In non-LDL-reactive clones, V-segment usage was not restricted [135]. Antibodies against the predominating V-chain segment TCRBV31 protected from atherosclerosis in vivo, likely by an elimination of atherosclerosis-relevant T cell clones. In a second study [91], the TCR clone containing the enriched β chain V-segment TRBV31 was used for a TCR-transgenic mouse, in which 90% of T cells expressed TRBV-31 and recognized LDL. In vivo, ApoB-specific CD4^+^ T cells developed into T_FH_ cells, activated B cells, stimulated the formation of germinal centers, and induced the expansion of plasma cells expressing anti-LDL immunoglobulins. Anti-LDL IgGs enhanced LDL clearance in the liver—a mechanism that led to decreasing LDL-C plasma levels and significantly smaller atherosclerotic lesions than in controls. Thus, ApoB-reactive T cells have the ability to serve as potent T_FH_. Whether the selection procedure in vivo used in this series of reports predisposes for a specific TCR/phenotype that does not predominate in TCR-WT mice is currently not known. Still, it remains an interesting speculation that low and high affinities between a peptide loaded MHC-II and the TCR induce distinct transcriptional programs in T cells, which may induce distinct T_H_ types as recently suggested [221].

## 5. Function of ApoB-Specific CD4^+^ T Cells

As stated above, LDL-C levels correlate with adverse clinical outcomes [1] and the progression of coronary atherosclerosis [5] and represent one of the best established targets for medical prevention of CVD [223]. Besides the plethora of innate-related inflammatory mechanisms, LDL-C likely serves as autoantigen in the atherosclerotic plaque, which is best demonstrated by the ability of human plaque T cells to secrete pro-inflammatory cytokines and proliferate when restimulated with LDL preparations—an effect highly dependent on MHC-II antigen presentation, suggesting specificity of these results [22,135]. In addition, ApoB-specific T_H_ cells have been detected in humans by MHC-II tetramers [118]. The function of ApoB-specific CD4^+^ T cells in humans can currently only be inferred from their differentiation into classical T_H_ types of immunity. These findings suggest that in the presence of sub-clinical or clinical atherosclerosis, ApoB^+^ T cells in humans are more polarized towards T_H_17 and T_H_1 cells, while only maintaining a residual T_reg_ signature in healthy individuals. It is therefore plausible that the compartment of ApoB-specific autoreactive T_H_ cells may have protective properties in health but switch into pro-inflammatory T_H_ types in disease. Whether this switch is causal for the pro-inflammatory environment in the plaque or a result of the inflammatory response that accompanies atherosclerotic disease is currently unknown. 

**Figure 2:**
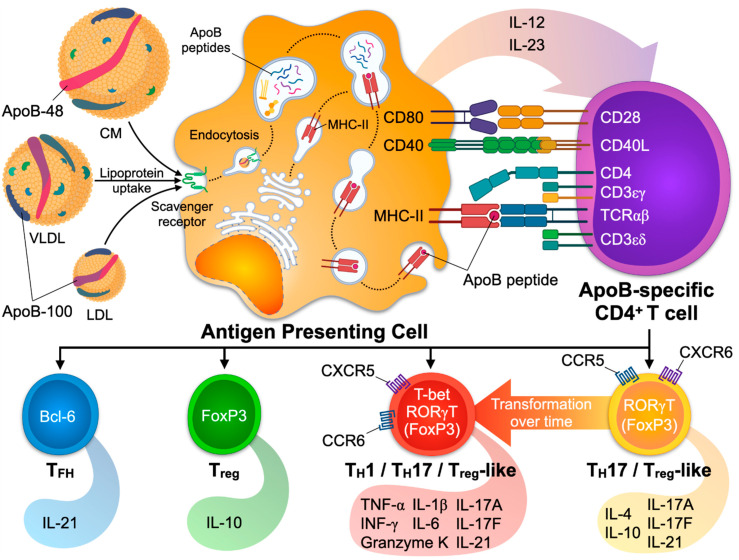
Activation and differentiation of ApoB-specific CD4^+^ T cells in mice. The ApoB-containing apolipoproteins LDL, VLDL, and chylomicrons (CM) are taken up by antigen-presenting cells (APCs) by endocytosis. After their intracellular processing, apolipoprotein-derived peptides are loaded on MHC-II molecules, before the entire MHC-II-peptide complex is transposed to the cell membrane. There, MHC-II-peptide complexes can be recognized and bound by a specific T cell receptor (TCR). In combination with sufficient co-stimulatory signaling events provided by the APC, a naïve CD4^+^ T cell is activated and may differentiate into distinct, partially overlapping T_H_-types of immunity: Most ApoB-specific CD4^+^ T cells (ApoB^+^) express transcriptomes and markers of T_H_17 and T-regulatory cells (T_reg_). They express CCR5 and CXCR6, two known chemokine receptors (CCRs) required for aortic homing. Over time, ApoB^+^ cells acquire additional pro-inflammatory transcriptional programs and express the T_H_1 transcription factor T-bet, as well as the CCRs CXCR5 and CCR6. The initially detectable protective T_reg_ signature is lost in this process. After vaccination, IL-10 secreting FoxP3^+^ApoB^+^ cells have been described at the site of vaccination. In transgenic mice, only expressing a TCR that recognizes a specific ApoB-peptide, a part of ApoB^+^ cells differentiates into T_FH_ that promote plasma cells generation and the production of LDL-lowering anti-LDL antibodies.

Since 1959, when Gero et al. performed the first atheroprotective vaccination of rabbits with LDL [224], numerous studies in rodents have demonstrated that vaccination with either native, modified (oxidized) LDL, or (peptides from) ApoB has the potential to elicit a T-cellular immune response that prevents atherosclerosis [225,226,227,228,229,230,231,232,233,234]. ApoB-100 and its truncated version, ApoB-48, which is present in chylomicrons [235], contain several immunogenic T cell peptide epitopes. In contrast, B cell epitopes are mostly located on lipid moieties of native and modified apolipoproteins [23]. Direct immunization with ApoB-100 and ApoB-100 peptides protects from atherosclerosis, likely by the induction of IL-10 secreting protective T_regs_ [199,236,237,238]. The immunogenic peptide epitopes from human or mouse ApoB that have been validated by vaccination of mice expressing the wildtype, mouse ApoB-100, or human transgenic ApoB-100 include the peptides p3, p6, p18, p101, p102, p103, p210, p265, and p295 [49,239,240,241]. p18 is the only so far identified peptide that is sequence-identical in mouse and human ApoB. The ApoB-peptide p6 (ApoB_978–993_, sequence TGAYSNASSTESASY) has been most extensively characterized. p6 is located in the surface region of ApoB-48 and ApoB-100 at the interface of the amphipathic core region [240]. Vaccination with p6 induces an antigen-specific T cell response with cellular proliferation and cytokine secretion [240]. Interestingly, ApoB p6-reactive T helper cells isolated from immunized mice and transferred to donor mice promoted atherosclerosis in abdominal aortas [242], while vaccination with p6 prevented atherosclerosis in *Apoe*^−/−^ mice in another report [240]. This finding is striking because most reports employing vaccination with ApoB/LDL have suggested a primarily protective phenotype encompassing T_regs_ in the atherosclerotic aorta and spleen as well as IL-10 secretion [240,243,244] or a decrease of T_H_1 immunity [245]. Notably, some of these favorable effects were abolished by a depletion of T_regs_ [245]. The functional dichotomy raised by vaccination studies using p6 was partially clarified in later studies that took use of a tetramer of MHC-II loaded with the peptide p6. It was suggested that ApoB-specific CD4^+^ T cells stem from a T_reg_-like T_H_17 cell with a partially protective phenotype that was lost during progressing natural disease and replaced by a T_H_1-like phenotype with several pro-inflammatory transcriptional programs including TNF-α, IL-6, and IFN-γ (Figure 2). These findings are consistent with the phenotypes observed in human ApoB restimulation assays and suggest that ApoB-specific CD4^+^ T cells per se are neither atheroprotective nor pro-atherogenic. Instead, phenotypes of ApoB-reactive CD4^+^ T cells may be dictated by the microenvironment in the plaque or systemic inflammation [49]. A protective role of antigen-specific CD4^+^ T cells can also be derived from the observation that a genetic knock-out for MHC-II, which abrogates antigen-recognition and -presentation, promotes de novo atherosclerosis [246,247]. Because hypercholesterolemia (with elevated LDL-C levels) in mice favors the differentiation of T_regs_ in the early stages of atherosclerosis [60,61] and enhances TCR-signaling events in T_regs_ [59], it must be hypothesized that in healthy mice, a cellular T_reg_-driven protective autoimmune response against LDL-C/ApoB exists [247]. It is therefore plausible, but remains experimentally unproven, that early ApoB-reactive T_regs_ have the ability to prevent atherosclerosis. It is also tempting to speculate how these cells are generated and why they appear even in healthy individuals and mice. The general concept of autoimmunity states that the immune system distinguishes between self and non-self [248]. Some CD4^+^ T cells expressing a T cell receptor (TCR) that recognizes self-peptides loaded on MHC-II with a high affinity are eliminated by negative selection [249,250]. However, negative selection is not very efficient [251,252] as demonstrated by the existence of self-antigen specific CD4^+^ T cells in a naïve organism [181]. A part of these surviving T cells develops into pathogenic T_eff_, while these with a low to intermediate affinity turn into protective T_regs_ [253,254]. Thus, autoimmunity is understood as a competition of protective and pathogenic CD4^+^ T cells that both recognize (different) self-peptides from the same autoantigen. Indeed, several reports have demonstrated the existence of autoreactive T-helper cells with a protective T_reg_ and a pathogenic T_eff_ phenotype in autoimmune disease of the central nervous system, graft-versus-host-disease, and in type 1 diabetes [124,126,255,256,257,258]. During development of disease, this fine-tuned balance is thought to shift towards a relative overrepresentation of pathogenic, often T_H_1 T cell phenotypes. In atherosclerosis, autoreactive CD4^+^ T cells with a predominant or partial T_reg_ phenotype in the absence of atherosclerosis and a pathogenic phenotype in established disease have been found in two studies employing MHC-II tetramers in humans and mice [49,118]. Whether pathogenic T_eff_ exclusively develop from switching T_regs_ or independently and how the composition of autoreactive CD4^+^ T cells with T_reg_ and T_eff_ cells changes over time, is currently unknown. It also remains unclear if the phenotypic switch of ApoB^+^ T cells to a more pathogenic phenotype is a cause or a consequence of exaggerated inflammation in advanced atherosclerosis. Whether the resulting T_H_1-like ApoB-reactive T cells are in fact pro-atherogenic has not been directly tested. TF expression from human ApoB-reactive T cells suggests that FoxP3 protein-expressing T_regs_ dominate the phenotypic repertoire of ApoB-specific T_H_ cells in healthy humans. In healthy mice, only a minor fraction of ApoB-specific CD4^+^ T cells expresses FoxP3, while the majority shows a transcriptional similarity to T_regs_ but does not express FoxP3 protein [49]. Lineage-tracing experiments have demonstrated that not all FoxP3^neg^ ApoB-specific T cells stem from initial FoxP3^+^ T_regs_ [49]. Therefore, T_reg_ plasticity does not explain the generation of all pathogenic ApoB-specific T_eff_ cells alone [20]. Notably, mouse ApoB^+^ T cells demonstrate at least a partial T_FH_ signature with *Bcl6* and *Cxcr5* transcripts [49], indicating that the T_FH_ phenotype observed in the ApoB-TCR-transgenic mouse may represent a natural occurring phenotype, albeit likely overrepresented in the experimental settings in the transgenic mouse model [91]. Potential factors that cause ApoB-reactive T cells to transform from protective (T_reg_-like) to pathogenic T_H_-types have not been explicitly investigated and are currently unknown. However, it is plausible that factors that influence the stability of T_regs_—hypercholesterolemia, inflammatory cytokines, local hypoxia, and changes in cellular metabolism [20,25,117]—partially overlap with those favoring the pathogenic conversion of functionally protective FoxP3^neg^ ApoB-specific CD4^+^ T cells. 

## 6. Clinical Translation and Outlook

The fundamental role of inflammation in atherosclerosis has been increasingly appreciated in the last decades [20,259]. Clinical landmark trials, such as the CANTOS and COLCOT trials [17,18,19], have highlighted the potency of anti-inflammatory treatment strategies in cardiovascular disease prevention. However, the limitations of unspecific anti-inflammatory treatments remain considerable as evidenced by increased rates of infection and missing experience on long-term treatments. By contrast, the development of antigen-specific immunomodulation holds the promise of specific antigen-directed therapies with only minimal side-effects [234]. The recent development of technologies to detect ApoB-specific T cells at the single cell level, including MHC-class II tetramers, has greatly widened our understanding of adaptive immune mechanisms in atherosclerosis [25]. It is now clear that ApoB-specific T_H_ cells exist in mice and humans. These undergo dramatic transcriptional, numeric, and phenotypic changes throughout the natural course of atherosclerosis. While the predominant pro-atherogenic T_H_1 phenotype of ApoB-reactive T_H_ cells in the blood of patients with advanced atherosclerosis and in atherosclerotic plaques is consistent with older findings, it is striking that ApoB-reactive T cells in early disease are transcriptionally closer to immunosuppressive T_regs_ [49,118]. This observation provides a reasonable explanation for enhanced numbers of ApoB-specific T_regs_ in numerous mouse vaccination studies and suggests that autoimmunity per se is not detrimental but required to restrain a pathogenic immune response in most healthy individuals [247]. Vaccination with tolerogenic adjuvants and immunogenic ApoB-peptides may therefore have the potential to reinforce the protective limb of ApoB-specific immunity even in patients with established atherosclerosis. The successful implementation of a human atherosclerosis vaccine in clinical practice will depend on several developments that have yet to be made: First, exact doses, routes of delivery, and adjuvants in a vaccine need to be clarified. In addition, additional autoantigens beyond ApoB are likely to exist and may be found in a screening of peptides that are naturally presented on MHC-II in atherosclerotic plaques. Second, patients with an immune-mediated type of atherosclerosis independent of an enhanced diabetes-, lipid-, inflammation-, or thrombosis-associated risk [260] will have to be identified. Third, biomarkers will have to be developed that are suitable for assessing the efficacy of vaccination. While antibody-titers are usually employed to screen vaccination efficiency, it is not clear if ApoB-peptides located within the inner core of LDL-C and other lipoprotein particles will elicit both cellular and humoral immune responses. It is now evident that different types of atherosclerosis-associated risk exist in humans. For instance, certain risk factors associate with different manifestations of atherosclerotic disease, such as evidenced by smoking and the enhanced prevalence for peripheral arterial disease (PAD) [261,262]. Under optimal lipid-lowering therapies, a residual inflammatory risk remains [263] and even with lipid levels in the desired or below target range and in the absence of residual inflammation, event-rates remain high. It may therefore be speculated that a proportion of this excessive, currently not addressable risk is related to autoimmune mechanisms. Notably, even with LDL-C levels at or below the recommend target range of 40 to 55mg/dL [223], LDL-C is not entirely depleted, and this low, but chronic abundance of an autoantigen may suffice to induce pathogenic anti-LDL/ApoB immunity. The possible existence of an independent atherosclerotic immune risk is also justified by the clinical association of chronic immune and atherosclerotic disease [264] in otherwise healthy individuals. Assays that allow the quantification of ApoB-specific T cells, such as by restimulation or tetramers, will be of great use to quantify such immune-risk in future clinical practice. 

## Figures and Tables

**Table 1:** CD4^+^ T cell subsets and functions in atherosclerosis.

Lineage	TF	Effector Cytokines	Role in Mouse Atherosclerosis	Regulation in Human Atherosclerosis
T_H_1	T-bet	INF-γ, IL-2, IL-3, IL-6, TNF-α, lymphotoxin	Pro-atherogenic [37,39,40,41,42,43,44]	T_H_1 dominance in atherosclerotic lesions [35,36], higher IFN-γ plasma levels in patients with CAD [127], higher IL-6 and TNF-α plasma levels in patients with MI [128].
T_H_2	GATA3	IL-4, IL-5, IL-10, IL-13	Pro-atherogenic [79,82] Atheroprotective [80] No effect [81]	Lower T_H_2 cell numbers and decreased IL-4 secretion by CD4^+^ in patients with MI [83], lower IL-5 plasma levels in patients with subclinical atherosclerosis [84]
T_H_9	FoxO1, BATF, IRF4	IL-9	Pro-atherogenic [107]	Higher IL-9 plasma levels in patients with atherosclerosis and ACS [105] Unchanged T_H_9 numbers in patients with ACS [106]
T_H_17	RORγT	IL-17A, IL-17-F, IL-21, IL-22	Pro-atherogenic [64,65,66,67] Atheroprotective [70,71,72,73,74] No effect [76]	Higher IL-17 plasma levels in patients with unstable angina or MI [68,69], lower IL-17 plasma levels in patients with MI [75], unchanged IL-17 plasma levels patients in patients with CAD [77]
T_H_22	AHR	IL-22	Pro-atherogenic [109]	Higher T_H_22 cell counts and IL-22 plasma level in patient with an ACS [106,110]
T_reg_	FoxP3, CD25	IL-10, TGF-β	Atheroprotective [54,55,56]	Lower T_reg_ numbers in blood from patients with MI [57] and ACS [68,69], low T_reg_ numbers predict MI [58], higher T_reg_ numbers in blood of patients with CAD [49]
T_FH_	Bcl6	IL-21	Pro-atherogenic [50,87,88]	Higher T_FH_ count in patients with advanced atherosclerosis [90]
CD4^+^ CTL		TNF-α, INF-γ, perforin, granzyme A, B	Not present in mice	Higher numbers in blood from patients with ACS [95] and with end-stage renal disease and atherosclerosis [129], enrichment in unstable atherosclerotic lesions [97,98,99]
NK T cells		Multiple, including perforin and granzymes	Controversial [113]	Accumulation of NKT cells in rupture-prone atherosclerotic plaques [112].

TF, transcription factor; T_H_, T-helper; T_reg_, T regulatory cell; T_FH_, T follicular helper cell; AHR, aryl hydrocarbon receptor; MI, Myocardial Infarction; ACS, Acute Coronary Syndrome; CAD, Coronary Artery Disease; CTL, cytotoxic lymphocyte; NK, natural killer.
